# Investigation of characteristics as endodontic sealer of novel experimental elastin-like polypeptide-based mineral trioxide aggregate

**DOI:** 10.1038/s41598-021-90033-9

**Published:** 2021-05-18

**Authors:** Hyun-Jung Kim, Ji-Hyun Jang, Sun-Young Kim

**Affiliations:** 1 Department of Conservative Dentistry, Kyung Hee University Dental Hospital, Seoul, South Korea; 2 Department of Conservative Dentistry, School of Dentistry, Kyung Hee University, Seoul, South Korea; 3 Department of Conservative Dentistry and Dental Research Institute, School of Dentistry, Seoul National University, 101 Daehak-ro, Jongno-gu, Seoul, 03080 South Korea

**Keywords:** Calcium-based cement, Dental biomaterials, Mineral trioxide aggregate, Endodontics

## Abstract

Although mineral trioxide aggregates (MTA) have been adopted as an endodontic sealer because of excellent sealing effect and bioactive property and been modified with improvement of its characteristics, the developed MTA sealers have not yet satisfied all the ideal requirements of endodontic sealers. The aim of this study was to assess the characteristics of elastin-like polypeptide (ELP)-incorporated MTA for use as an endodontic sealer and compare them with those of commercial MTA sealers. Two commercial MTA sealers and three experimental ELP-incorporated MTA sealers with 0.3, 0.4, and 0.5 liquid/powder (L/P) ratio for 10 wt% ELP liquid were evaluated. The push-out bond strength, flow rate, sealer penetrability and wash-out resistance were tested and the sealer-dentin interface was observed using a scanning electron microscope (SEM). Our study revealed the ELP-incorporated MTA sealer, especially in 0.4 L/P ratio, exhibited the higher push-out bond strength and flow rate (P < 0.05), and equal or superior sealer penetration and remarkable wash-out resistance compared to commercial MTA sealers. The groups of ELP-based experimental sealers also exhibited more intimate contact with dentin compared to the commercial MTA sealers. Our research will suggest the possible adoption of the ELP-incorporated MTA as endodontic sealer for clinical use.

## Introduction

The final step of endodontic treatment is to fill the root canal in order to achieve hermetic sealing of the root canal system, preventing oral bacteria from re-entering and colonizing the root canal^1^. Root canals are generally obturated with combinations of core filling materials, i.e. gutta percha, and endodontic sealer. An endodontic sealer fills up the void between the core filling materials and root canal wall, thus providing a fluid-tight seal. Extrusion of endodontic sealers beyond the apical foramen may lead to direct contact with the soft tissue at the apex of the root canal system, which can affect the healing of periapical tissue^2,3^. Accordingly, biocompatibility forms an important requisite among the ideal properties of a sealer^4,5^. For this reason, the endodontic sealers based on mineral trioxide aggregates (MTA) which show excellent biocompatibility and antimicrobial effect, have garnered great attention in dentistry^6^. Several MTA-based sealers have been recently developed and are now used in endodontic treatment^7,8^.

Despite its good biocompatibility and sealing ability, MTA has low adhesion to hard tissue, low washout resistance due to slow setting time, and difficulty in handling^9,10^. Thus, current MTA-based sealers include the additives to improve the working time, solubility, and handling properties^6,10^. For example, calcium sulfate was added to increase the working time of MTA-based sealer^11,12^ and calcium chloride was used to reduce the long setting time and solubility of MTA sealer^10,11,13^. Propylene glycol increased the flow rate and a synthesized water-soluble polymer improved handling properties of MTA-based sealer^7,14,15^. Although the use of MTA-based sealers is increasing in endodontic treatment with favorable outcomes as shown in the previous studies, there is still plenty of scope for developing better MTA-based sealers^16,17^. Specifically, there is still insufficient information on whether the current MTA-based sealers meet the required characteristics for endodontic sealers, such as the ability to adhere to the root canal wall, flow rate, and washout resistance in tissue fluids.

Elastin-like polypeptides (ELP) are synthesized biopolymers inspired by human tropoelastin and are now widely used as drug carriers for cancer therapy, tissue engineering, and protein purification^18^. The structure of ELP is composed of five repeated amino acids, valine–proline–glycine–Xaa–glycine (where the “guest residue” Xaa is any amino acid except proline)^19^. An ELP named V125E8, which is a 125 times-repeated structure of a valine–proline–glycine–valine–glycine unit with eight glutamates added on C-terminal, has been recently investigated as a form of mixture with MTA for dental application^20–22^. Previous studies have revealed several advantages of the ELP-MTA mixture, such as enhanced mechanical properties, washout resistance properties, more intimate contact with the dentin wall, good flow rate, and increased adhesion without compromising the biocompatibility of MTA^20,22^.

Based on the aforementioned advantages of supplementing ELP with MTA, we realized that V125E8-incorporated MTA could be employed as an endodontic sealer to fulfill the requirements. Therefore, we aimed to investigate the characteristics of the endodontic sealer of experimental ELP-supplemented MTA mixtures with various ratios of ELP and MTA and compare them with those of commercial MTA sealers. This study examined the bond strength to root dentin, flow rate, penetrability, and washout resistance of experimental ELP-MTA sealers and commercial MTA sealers.

## Materials and methods

### Experimental groups and materials used

Five groups of sealers were evaluated in this study: Group BE, BrightEndo MTA sealer (Genoss, Suwon, Korea); Group ES, Endoseal MTA (Maruchi, Wonju, Korea); Group 03ELP, experimental ELP-based MTA sealer of 0.3 Liquid/Powder (L/P) ratio; Group 04ELP, experimental ELP-based MTA sealer of 0.4 L/P ratio; Group 05ELP, experimental ELP-based MTA sealer of 0.5 L/P ratio. The first two products (BE and ES) are commercially available. For the experimental ELP-based MTA sealers, we synthesized an ELP, V125E8, as described in a previous study^23^.

The name V125E8 means 125 repetitions of the amino acid sequence of VPGVG with the addition of an E8 functional group at the C-terminal^23^. A brief description of the procedure of synthesis was as follows: gene editing by annealing and ligation of synthetic oligonucleotides (IDT Inc., Coralville, IA, USA), plasmid transforming into BLR (DE3) *E. coli* (EMD Millipore, Gibbstown, NJ, USA), E. coli culture, and purification using the inverse transition cycling method^24^. V125E8 was supplemented as a 10 wt% liquid form to make a mixture of MTA (Proroot MTA; Dentsply Tulsa Dental, Tulsa, OK, USA) with various L/P ratios from 0.3 to 0.5, for the experimental ELP-based MTA sealers used in this study. It was chosen because it improved the mechanical and bonding properties of calcium silicate cement in our previous studies^22,25^. The composition of commercial MTA sealers and MTA for experimental sealers is presented in Table 1.

**Table 1:** Composition of the commercial MTA sealers and the MTA tested in this study.

Commercial brand	Manufacturer	Composition
BrightEndo	Genoss, Suwon, Korea	Calcium silicates, calcium sulfates, zirconium oxide, bismuth oxide, methyl cellulose, N-methyl-2-pyrrolidone
Endoseal	Maruchi, Wonju, Korea	Calcium silicates, calcium aluminates, calcium aluminoferrite, calcium sulfates
Proroot MTA	Dentsply, Tulsa, OK, USA	Tricalcium silicate, dicalcium silicate, tricalcium aluminate, calcium sulfate, bismuth oxide

### Push-out bond strength test

Human single-rooted premolars with one canal, either intact or containing only small carious lesions, were selected for this study (n = 10 per group). The included teeth were obtained from patients, whose teeth were indicated for extraction due to orthodontic reasons, as approved by the Kyung Hee University Institutional Review Board (KHU-1808-1), and all methods were performed in accordance with the relevant guidelines and regulations. Informed consent was obtained from all participants included in the study. The teeth were decoronated at the level of cemento-enamel junction, using a high-speed diamond saw (IsoMet 5000, Buehler, Lake Bluff, IL, USA) to obtain a root length of 16 mm. Working length was established using a size 10K-file (Mani Inc., Tochigi, Japan) to the root canal terminus and subtracting 0.5 mm from this measurement.

Fifty root canals were prepared using K3XF (SybronEndo, Orange, CA) rotary nickel-titanium files up to size 45 with 0.06 taper. Irrigation was performed with 5.25% NaOCl solution in a 5 mL disposable plastic syringe (Ultradent Products Inc., South Jordan, UT, USA) with a side-ventilation tip (Ultradent) placed passively into the canal, up to 1 mm from the apical foramen. After root canal preparation, the canals were rinsed with 1 mL of ethylenediaminetetraacetic acid (EDTA) to remove the smear layer on root dentin and then with 5.25% NaOCl solution as the final irrigant. Root canals were dried with absorbent paper points (Meta Biomed, Chungbuk, Korea) and obturated only with the sealer, using a syringe. Commercial MTA sealers (Groups BE and ES) were handled according to the manufacturer’s instructions. Experimental sealers (Groups 03ELP, 04ELP, and 05ELP) were introduced into the canal for obturation, using a 19-gauge needle tube and an E/Z syringe (AccuDose, Centrix, Shelton, CT, USA). Every sealer was introduced into the canal until completely filled. Canal-filled roots were checked radiographically and then stored at 100% humidity and 37 °C for 1 week.

Canal-filled teeth were horizontally cut into 1 mm-thick sections perpendicular to the root canal, using a water-cooled high-speed diamond blade (IsoMet 5000). Two 1 mm-thick sections of the mid-root were selected from each tooth. Prior to the push-out test, the major (*DL*) and minor diameter (*DS*) of oval-shaped cavities were measured with a digital caliper (Mitutoyo Corp, Kanogawa, Japan) and recorded.

The specimens were mounted with apical side up and coronal side down to avoid interference due to root canal tapering. The push-out bond strength was measured using a universal testing machine (AGS-X, Shimadzu, Tokyo, Japan) at a crosshead speed of 1.0 mm/min with a stainless steel plunger 0.7 mm in diameter (n = 20)^26^. The maximum load applied to the specimens was recorded in Newtons before dislodgement occurred. To express the bond strength in MPa, the recorded value in Newtons was divided by area in mm^2^ and calculated using the following formula:

$$Area\; \left(\text{mm}^{2}\right)=\pi \times \frac{(DL+DS)}{2}\times h,$$

where $\pi$ is the constant 3.14, *DL* is the major diameter, *DS* is the minor diameter of the oval shaped cavity, and $h$ is the thickness of the dentin disc in millimeters. Naïve formula was adopted for the calculation of perimeter of ellipse for oval canal^27^.

The failure modes of debonded samples were not counted because all showed MTA remnants on the cavity wall surface, which indicates mixed failure with both adhesive failure between dentin and MTA sealer and cohesive failure in MTA sealer according to the previous study^25^.

### Flow rate measurement

The flow rate of the sealers was tested with a modification of the ISO 6876 criteria^28^. Briefly, a 0.5 mL of each sealer was placed on the center of a glass plate with dimensions of 70 × 70 × 8 mm^3^. After 3 min, a 100 g glass plate was placed on top of the material. The load was removed 10 min after the start of mixing, and the minimum and maximum diameters of the spread sample disks were measured with a digital caliper (Mitutoyo Corp) with a resolution of 0.01 mm. The mean values were then calculated. If the disk was not uniformly circular (if the difference between maximum and minimum diameters was not within 1 mm), the test was repeated. Flow rates were measured thrice for each group.

### Penetrability test

Extracted human single-rooted premolars were obtained (n = 5 per group). The canal preparation procedure was identical to that of the push-out bond strength test.

For canal-filling of the prepared canal, main gutta-percha (GP) cone with # 40 or 45, 0.06 taper were used. The master cone was checked in the canal prior to placement by noting the point where “tug-back” at working length was first achieved. To allow penetrability analysis, each sealer was labeled with Rhodamine B (Sigma-Aldrich, St. Louis, USA) to an approximate concentration of 0.1 wt%. The sealers of Groups BE and ES were used according to the manufacturer’s instructions. After the sealer was injected, the master cone was inserted with a couple of pumping actions. Experimental ELP-based MTA sealers were introduced into the canal for filling within 3 min after mixing, using a 19-gauge needle tube and an E/Z syringe (AccuDose) and the master cone was inserted with a couple of pumping actions. Root canal filling was performed with the continuous wave technique using Duo-alpha (B&L tech, Seoul, Korea) and Duo-beta (B&L tech). Canal-filling was confirmed radiographically and the samples were then stored at 100% humidity and 37 °C for 1 week. Root canal filled teeth were embedded in acrylic resin for cold mounting (MTDI, Daejeon, Korea), stored at ambient temperature for 24 h, and horizontally sectioned into 0.4 mm thickness using a water-cooled high-speed diamond blade (IsoMet 5000). Two sectioned specimens were selected from the middle third of the root from each tooth and its surfaces were serially polished with SiC papers (#400–4000 grit). The dentin segments were examined using an LSM700 confocal laser scanning microscope (CLSM) (Carl Zeiss, Oberkochen, Germany) (n = 10 per group). The absorption and emission wavelengths for rhodamine B were 540 nm and 590 nm, respectively. Each image was acquired in Z-stack mode using 30 sections with a 10–15 μm thickness of z-axis and tile scan mode to capture the whole surface of the specimens. Sealer penetration depth was measured at each segment on four different sites (mesial, distal, buccal, and lingual) using ImageJ software (version 1.8.0_172). At the same point of measurement, the length from canal wall to root surface was also measured. Thus, the percentage of sealer penetration was established^29^.

### Evaluation of dentin-sealer interface with SEM

Extracted human third molars were obtained (n = 2 per group). Dentin discs of 2 mm thickness were fabricated with a high-speed saw (IsoMet 5000). To evaluate the sealer–dentin interface in a closed cavity, a cylindrical cavity was prepared with a 2 mm diameter and 1 mm depth with one end open in each dentin disc. Each sealer was loaded into the cavities and then stored at 100% humidity and 37 °C for 1 week. Specimens were embedded in acrylic resin for cold mounting and were sectioned parallel to the long axis of the cavity using a water-cooled high-speed diamond blade (IsoMet 5000). The exposed internal surface was serially polished with SiC papers (#400–4000 grit). The smear particles were removed by soaking in 17% EDTA solution for 1 min. The specimens were dried at ambient temperature for 1 day and the dentin-sealer interface was then observed under SEM (JSM-840, JEOL Tokyo, Japan) after gold sputter coating.

### Evaluation of wash-out resistance property

BE and ES were injected on a petri dish to form a small disc of approximately 5 mm diameter with each syringe, and slightly tapping on the floor. Experimental ELP-based MTA sealers were freshly mixed with each L/P ratio and injected to form a small disc of approximately 5 mm diameter using a 19-gauge needle tube and an E/Z syringe (AccuDose, Centrix). N-2-hydroxyethylpiperazine-N′-2-ethanesulfonic acid (HEPES) solution (Sigma-Aldrich, St. Louis, MO, USA) at 37 °C was poured into the Petri dish and the sealer samples of all groups were observed at baseline, 5 min, 1 h, and 24 h in a 37 °C incubator^30,31^.

### Statistical analysis

Push-out bond strength (N), flow rate (mm), and percentage of sealer penetration were analyzed by one-way analysis of variance (ANOVA). The Bonferroni test was used for *post-hoc* analysis. The level of significance was set at α = 0.05. All statistical analyses were performed using SPSS 25.0.0.0 (IBM Corp., Armonk, NY).

## Results

### Push-out bond strength test

**Figure 1:**
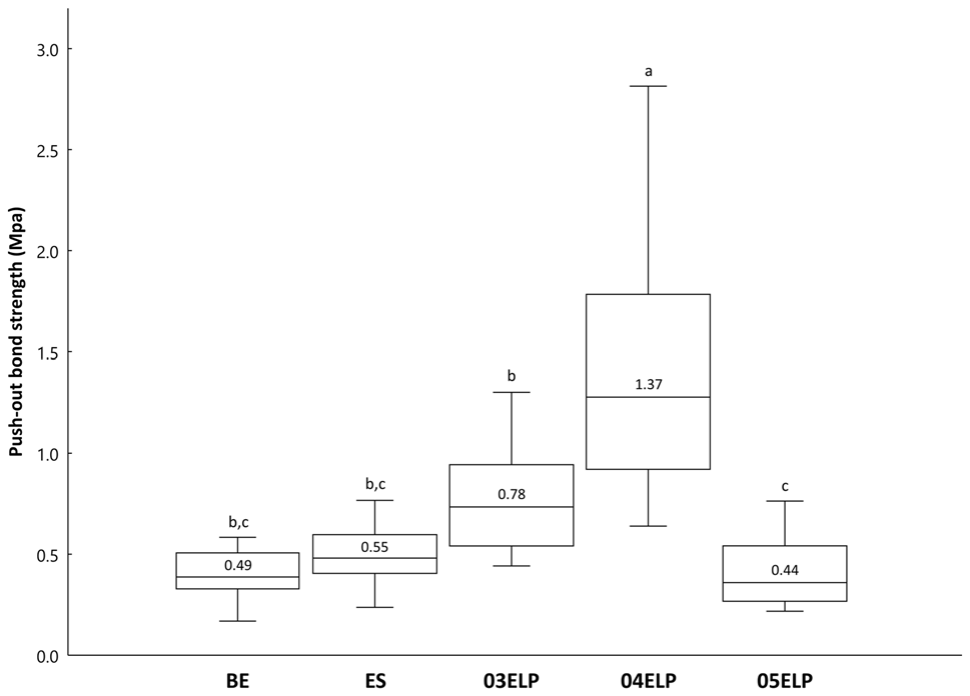
Push-out bond strength of the experimental groups. Different lower letters on top of the bar denote statistically significant differences between groups (P < 0.05).

The push-out bond strength data of all experimental groups are shown in Fig. 1. ANOVA revealed a significant difference among the experimental groups (P < 0.05). Group 04ELP showed the highest push-out bond strength among all the experimental groups (P < 0.05). Group 03ELP exhibited a significantly lower bond strength than Group 04ELP, whereas a significantly higher bond strength than Group 05ELP (P < 0.05). Groups BE and ES did not show a statistically significant difference from Group 03ELP and Group 05ELP, respectively (P > 0.05).

### Flow rate measurement

**Figure 2:**
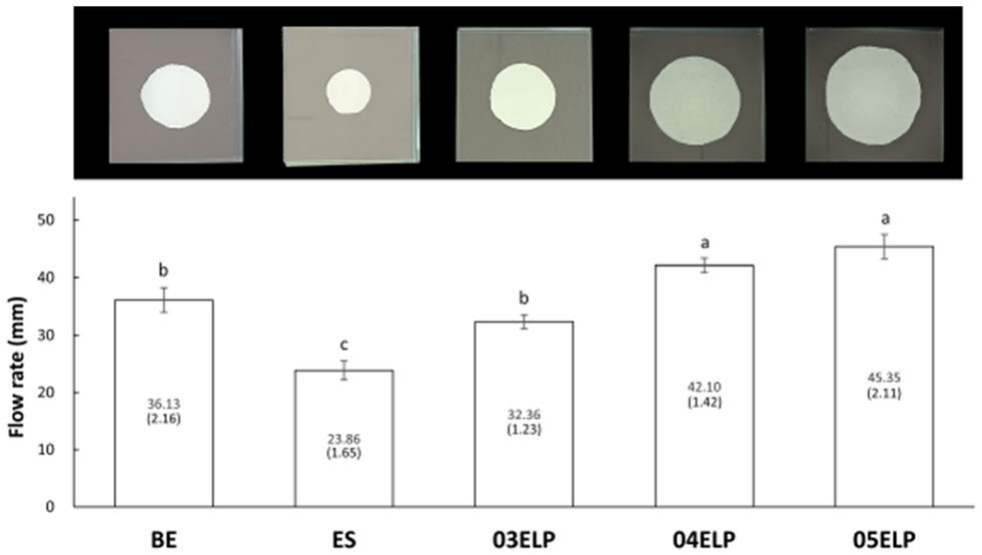
Representative images of flow test and mean flow rate of the experimental groups. Numbers in parentheses present standard deviation values. Different lower letters on top of the bar denote statistically significant differences between groups (P < 0.05).

The results of the flow rate measurement and representative images of each group are shown in Fig. 2. Groups 04ELP and 05ELP showed a significantly higher flow rate than the other groups (Groups BE, ES, and 03ELP; P < 0.05), and there was no significant difference between Groups 04ELP and 05ELP (P > 0.05). Group ES demonstrated the lowest flow rate among the groups (P < 0.05).

### Penetrability test

**Figure 3:**
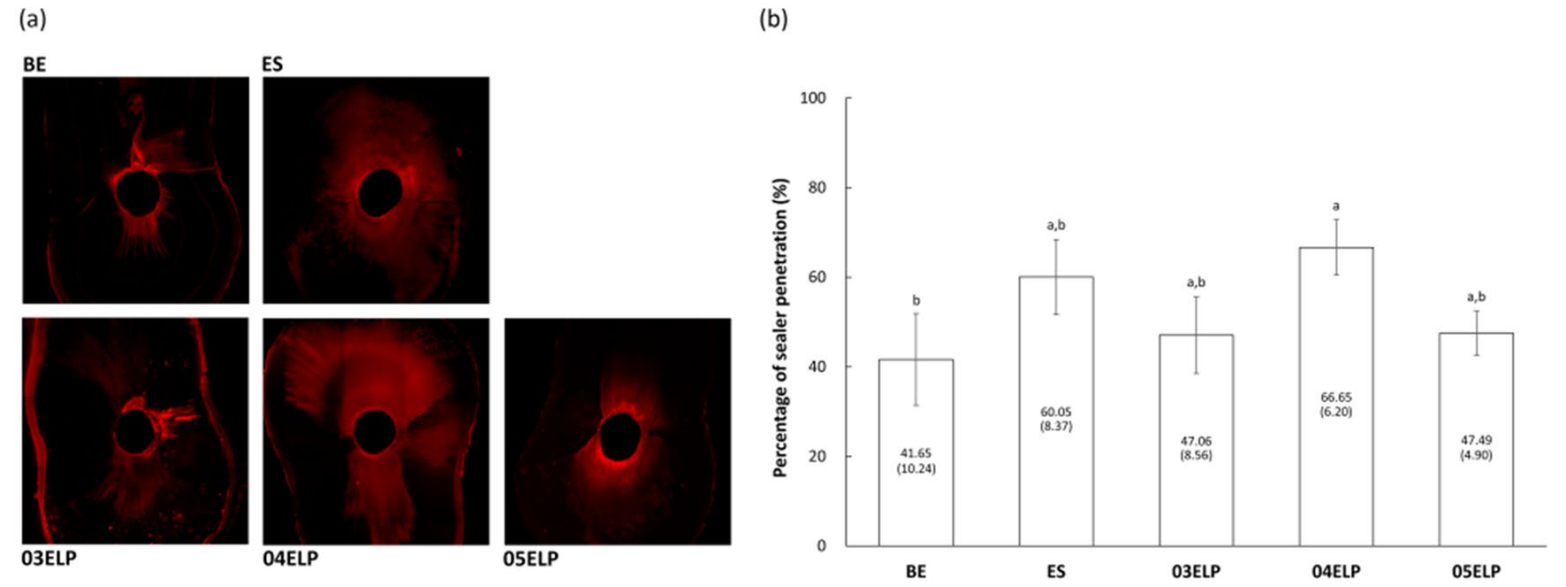
Sealer penetration level of the experimental groups. (**a**) Representative images of confocal laser scanning microscopic photograph (×10) showing the penetration of sealer to the dentinal tubules. (**b**) Mean percentage of sealer penetration. Numbers in parentheses present standard deviation values. Different lower letters on top of the bar denote statistically significant differences between groups (P < 0.05).

Figure 3a shows representative CLSM images for identification of sealer penetration. Penetration level of sealer was expressed as percentage (%), and the data are shown in Fig. 3b. Group 04ELP displayed a significantly higher penetration level than Group BE (P < 0.05), while there was no statistically significant difference between groups ES, 03ELP, and 05ELP (P > 0.05). In addition, Group BE showed no significant difference with Groups ES, 03ELP, and 05ELP (P > 0.05).

### Evaluation of dentin-sealer interface with SEM

**Figure 4:**
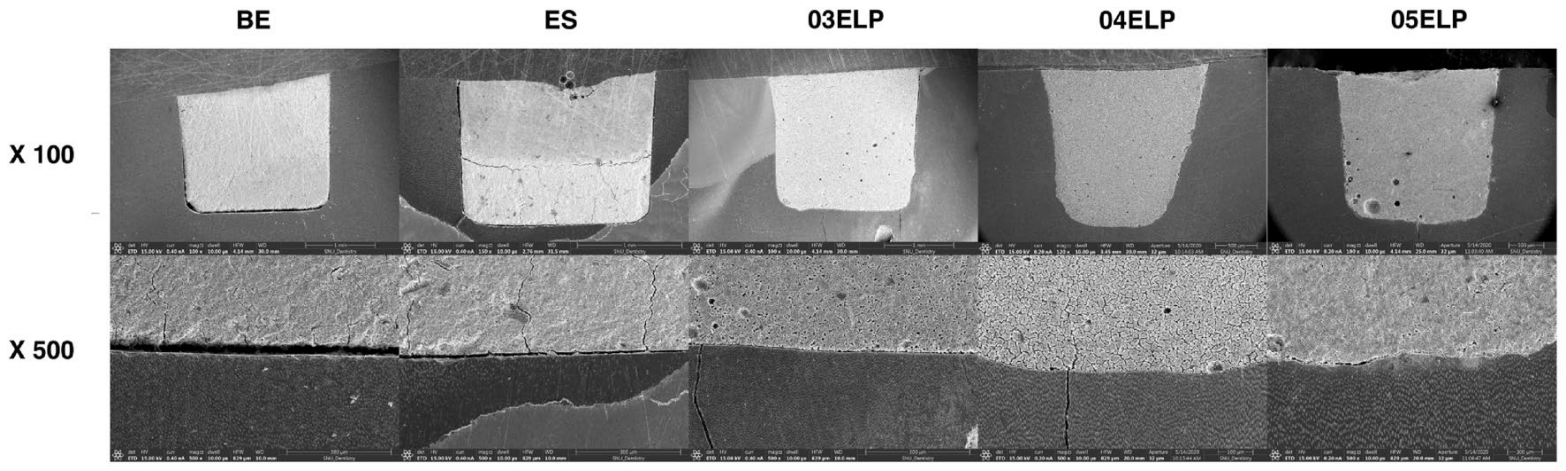
Representative SEM images of the dentin-sealer interface: low magnification (×100) of the experimental sealers in upper row and high magnification (×500) of the experimental sealers in lower row. ELP-based MTA sealers show more intimate contact with dentin compared to commercial MTA sealer groups.

Group BE demonstrated a compact sectional structure with few pores but a larger gap (approximately 25–30 µm) on the bottom compared to other groups (Fig. 4). The interfacial gap of Group ES was smaller (approximately 6–10 µm) at the bottom than Group BE but larger than that of the ELP-based MTA sealers. The groups of experimental ELP-based sealer exhibited relatively intimate contact with the dentin surface. Specifically, group 04ELP showed the best intimate contact with dentin surface compared to the other groups. The 05ELP group showed a relatively close interface, but more pores on the sectional structure.

### Evaluation of washout resistance property

**Figure 5:**
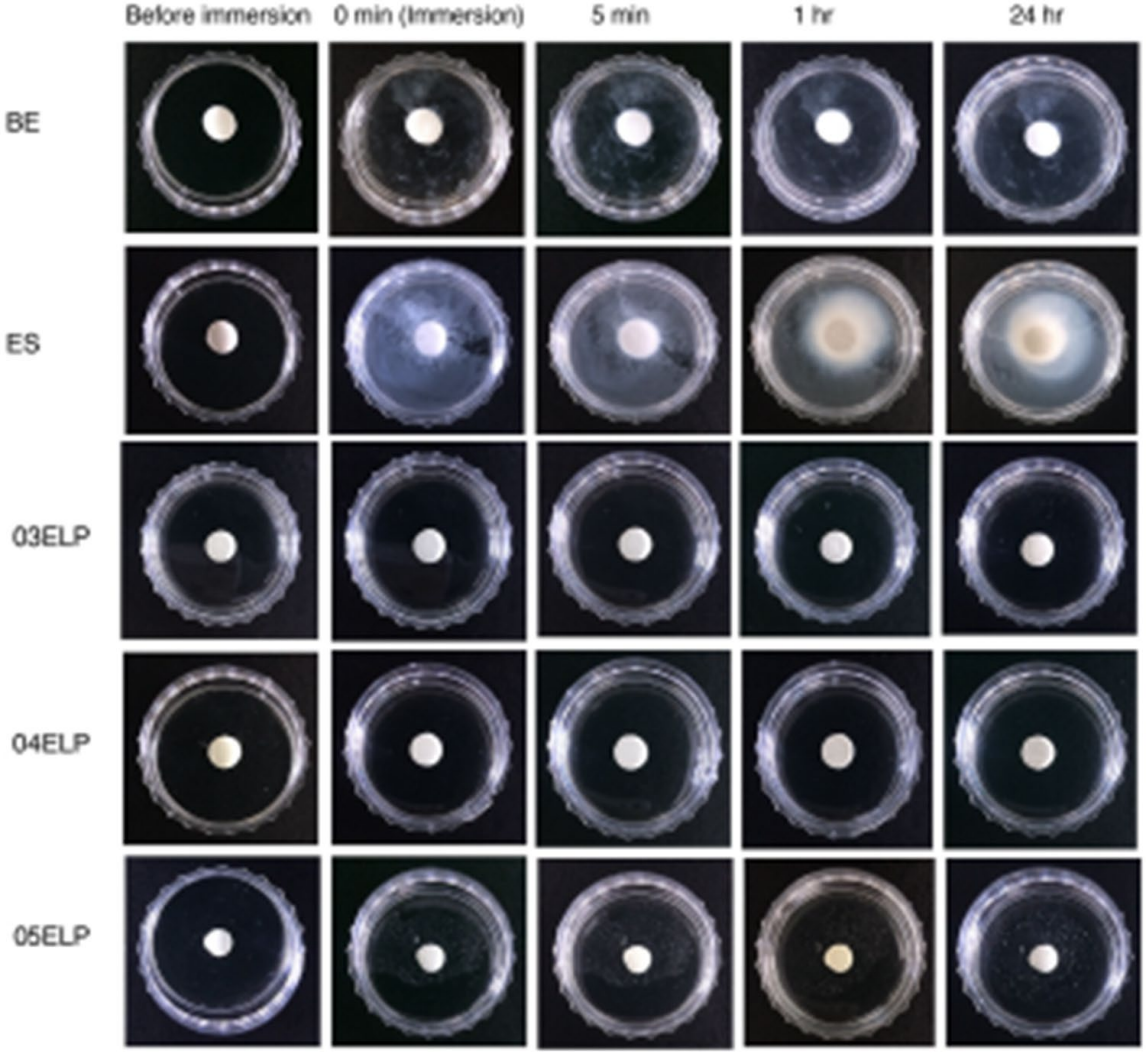
Evaluation of washout resistance. Groups 03ELP and 04ELP show distinct washout resistance than the other groups. ES demonstrates the least washout resistance among groups.

Group ES showed the highest washout among groups with a gradually increasing tendency during the 24 h observation period when compared to the other groups (Fig. 5). Group BE showed some degree of washout immediately upon immersion in HEPES solution, however, there was minimal increase in washout after 5 min. The experimental ELP-based MTA sealers generally exhibited a negligible amount of washout in HEPES solution compared to commercial MTA sealers (groups BE and ES). It was particularly difficult to find the washout particles in groups 03ELP and 04ELP during the 24 h observation period, whereas the 05ELP group exhibited slight washout immediately upon immersion in HEPES solution; however, there has been no significant change thereafter.

## Discussion

An ideal endodontic sealer requires several characteristics: adhesion to dentin preventing leakage from the interface between the core material and dentin, low viscosity, good wettability to flow into the irregularities on the walls of the root canal, good physicochemical properties and biocompatibility, and stimulating the reparative and biological sealing by mineralized tissue deposition in the apical foramen^12,32,33^. Most conventional root canal sealers have demonstrated inadequate biological activity and cytotoxicity in cultures, especially after fresh mix^34–36^. Ever since the development of MTA and its widespread use in dentistry, several studies have concluded that MTA can be used as an endodontic sealer because of its excellent sealing properties and biological properties^37,38^. Several modifications in the original formulation of MTA have been suggested in order to improve its characteristics as a root canal sealer; however, the developed MTA sealers have not yet satisfied all the ideal requirements^38,39^. In our previous studies, incorporation of ELP improved the physical properties of MTA in terms of compressive strength, bonding property, wettability and flow rate^20,22^. Considering these advantageous characteristics, we expected that ELP-incorporated MTA could be used as an endodontic sealer. Therefore, this study was designed to compare the physical properties of the ELP-based experimental MTA sealer with those of commercial MTA sealers, and to investigate the optimal proportion of ELP in experimental MTA sealer.

In the present study, the ELP-based experimental MTA sealer showed superior physical properties compared to commercial MTA sealers. In particular, the experimental sealer with 0.4 L/P ratio exhibited a higher push-out bond strength than the other groups (Fig. 1). V125E8 was selected as the functional additive ELP of the experimental sealer in this study. It has the octa-glutamic acid (E8) in the N-terminus, which is genetically constructed as a hydroxyapatite binding motif. The multi-glutamic acids make this peptide a negative charge, which increases the binding affinity to calcium ions on the dentin surface. This characteristic might have improved the push-out bond strength of the ELP-based experimental sealer. On the other hand, the 05ELP group showed significantly lower bond strength than groups 03ELP and 04ELP, and exhibited no significant difference from commercial MTA sealers. This might be due to the greater number of micro-bubbles in the fresh mixture of 05ELP compared to 03ELP and 04ELP. It is well known that more liquid generates more air bubbles in the mixtures^40^. In fact, more micro-bubbles were observed in the SEM samples of 05ELP compared to 03ELP and 04ELP in this study (Fig. 4).

Increased flow rate is one of the changes in the physical properties of freshly mixed MTA caused by ELP incorporation^22^. The amount of penetration through the dentinal tubules appeared to be unaffected by the increased flow rate. Group 05ELP presented lower penetration through the dentinal tubule, although it had the highest flow rate among groups. ES showed higher sealer penetration despite its lowest flow rate (Figs. 2 and 3). The penetrability of sealers through the tubules depends on many factors such as the presence of the smear layer, the chemical and physical properties of sealers, and root dentin permeability. Therefore, penetration of the sealer cannot be explained only by the flow rate. Among the ELP-incorporated MTA sealers, ELP proportion in MTA mixtures did not exhibit a direct relationship with penetration through the dentinal tubules. The 04ELP mixture showed better penetration through the dentinal tubules compared to 03ELP and 05ELP mixtures. This might be because the 03ELP mixture is too viscous and 05ELP has many micro-bubbles, which may have interfered with the penetration through the dentinal tubule despite its increased flow rate. ELP-based MTA sealers showed a small gap interface with dentin compared to commercial MTA sealers in this study (Fig. 4). The increased flow rate and chemical affinity to calcium ion of dentin hydroxyapatite may have caused more intimate contact with the dentin wall. The increased adhesion by V125E8 ELP incorporation revealed in our previous study is also considered to contribute to the enhanced contact with the dentin wall^22^. The optimally high flow rate prior to setting might help the endodontic sealer to have a stable adhesion, by the quality of sealer tag formed inside the dentinal tubule and filling up of the irregularities of the root canal wall.

The ELP-based experimental sealers with various L/P ratios showed a distinguished washout resistance compared to commercial MTA sealers used in this study. In particular, at 0.3 and 0.4 L/P ratio, a superior anti-washout property was observed (Fig. 5). This result might be caused by the reversible transition phase of V125E8 based on a transition temperature (Ts) between 31° and 33 °C. V125E8 exists in a non-aggregated and highly flowable form at ambient temperature below the Ts, while it shows aggregate form and viscous characteristics above the Ts^23^. In the washout resistance test, the testing temperature was set as the body temperature using a 37 °C HEPES buffer solution. At this temperature, the V125E8 solution exists in aggregate form, so that freshly mixed MTA particles are constrained in the aggregated peptide solution and would have shown notable washout resistance. Washout resistance is an important property among the requirements of an ideal sealer, because unset sealers may reach the periapical tissue beyond the apex due to flushing by body fluids, causing harmful effects on healing of inflamed periapical tissue, and breaking the hermetic seal in the apical foramen^32^. The excellent washout resistance of ELP-based MTA sealer is believed to be highly beneficial in preventing leakage and healing the apical tissue, especially, when it is set at body temperature.

In this study, the experimental ELP-based MTA sealers were compared with commercial MTA sealers for few characteristics, among the various requirements for endodontic sealer. More research is needed to investigate whether the ELP-based MTA sealer meets other ideal requirements. The experimental sealer contains polypeptides as a component, therefore, it is necessary to check the immune response of living tissue, although ELP is known to be biocompatible and less immunogenic^41^. In addition, in vivo tests and clinical trials will be necessary for clinical application of ELP-based MTA sealer.

Regarding the question of optimal ELP ratio in novel experimental MTA sealers, the 0.4 L/P ratio is considered to be the best among the three ratios in this study. 04ELP mixture showed the highest bond strength to dentin and presented a greater flow rate and penetration in the dentinal tubule. Specifically, 04ELP mixture exhibited remarkable washout resistance. 03ELP mixture showed higher performance in washout resistance, but lower performance in bond strength, flow rate, and penetration in dentinal tubules compared to 04ELP. 05ELP mixture showed less performance in most of the experimental outcomes of this study, except for presenting the best flow rate. Production of more air bubbles may also be another disadvantage in 05ELP mixture. The 0.4 L/P ratio might be an appropriate proportion to obtain the best properties for the requirement of an endodontic sealer.

## Conclusion

Within the limitations of this in vitro study, the experimental ELP-based MTA sealer, specifically at a 0.4 L/P ratio, exhibited better performance in bond strength to dentin, flow rate than other experimental groups. Also, it has the equal or superior quality in the aspect of dentinal tubule penetration, and remarkable washout resistance compared to commercial MTA sealers. Therefore, ELP-based MTA sealer might be adopted for clinical use after substantiating its properties with further studies and clinical trials. The incorporation of V125E8 into MTA may be an attractive strategy to formulate functionally advanced bioactive sealers in endodontic therapy and regenerative endodontics.
